# Prognostic significance of STING expression in solid tumor: a systematic review and meta-analysis

**DOI:** 10.3389/fonc.2023.1244962

**Published:** 2023-08-29

**Authors:** Younghoon Kim, Nam-Yun Cho, Lingyan Jin, Hye Yeong Jin, Gyeong Hoon Kang

**Affiliations:** ^1^ Department of Hospital Pathology, Seoul St. Mary’s Hospital, College of Medicine, The Catholic University of Korea, Seoul, Republic of Korea; ^2^ Laboratory of Epigenetics, Cancer Research Institute, Seoul National University College of Medicine, Seoul, Republic of Korea; ^3^ Department of Pathology, Seoul National University College of Medicine, Seoul, Republic of Korea

**Keywords:** meta-analysis, prognosis, STING, carcinoma, immunohistochemistry

## Abstract

**Objective:**

Stimulator of interferon genes (STING) is a key regulator in initiating innate immune response from sensing cytosolic DNA. Recent studies have revealed that the cGAS-STING signaling pathway has a crucial role in tumor development and progression across cancer types. Herein, we conducted a meta-analysis to explore the relationship between the immunoexpression of STING and the survival outcome of patients in various solid tumors. Studies relevant to the subject were searched from PubMed, Embase, and Web of Science.

**Results:**

Eleven studies including 2,345 patients were eligible for the analysis. STING expression in tumor cells was related to improved disease-free survival/recurrence-free survival (DFS/RFS) (HR = 0.656, 95% CI = 0.455–0.946, *p* = 0.024) but not with overall survival (OS) (HR = 0.779, 95% CI = 0.534–1.136, *p* = 0.194). STING expression in stromal cells, however, did not show significant correlation with DFS/RFS and OS (HR = 0.979, 95% CI = 0.565–1.697, *p*-value = 0.940 and HR = 1.295, 95% CI = 0.845–1.985, *p* = 0.235, respectively). In a subgroup analysis, STING expression in tumor cells was associated with better DFS (HR = 0.622, 95% CI = 0.428–0.903, *p* = 0.012). In tumor cells, favorable DFS/RFS were also related to studies from univariate analysis and the gastrointestinal system (HR = 0.667, 95% CI = 0.482–0.923, *p* = 0.015 and HR = 0.566, 95% CI = 0.330–0.971, *p* = 0.039).

**Conclusions:**

STING expression in tumor cells is associated with favorable outcome in solid tumors.

**Systematic review registration:**

https://www.crd.york.ac.uk/prospero/, registration number: CRD42023427027

## 1 Introduction

The ability of cancer cells to evade the immune system has been regarded as a crucial feature of tumorigenesis and tumor progression in human malignancies (1, 2). The cyclic GMP-AMP synthase (cGAS)-stimulator of interferon genes (STING) signaling pathway, responsible for sensing cytosolic double-strand DNA (dsDNA) and initiating innate immune response, has been considered as a potential driver of immune-mediated initiation, growth, and metastasis in cancer (3, 4). Accumulation of cytosolic DNA induced by DNA damage activates cGAS, leading to the production of cGAMP. cGAMP in tumor cells and antigen-presenting cells (APCs) activates STING, which triggers a cascade to recruit kinases IKK and TBK1 and leads to phosphorylation of IRF3 (5–8). Phosphorylated IRF3 acts as a transcription factor and mediates expression of immune-stimulated genes (ISGs), type 1 interferons (IFNs), and senescence-associated secretory phenotype (SASP) (3, 9, 10).

Cytokines released from tumor cells and APCs could activate cytotoxic CD8^+^ T cells and natural killer (NK) cells to facilitate tumor clearance (8, 11, 12). Furthermore, the activation of STING and its downstream cascade increases autophagy, induces senescence and chronic inflammation, and regulates differentiation of myeloid-derived suppressor cells (MDSCs) and tumor-associated macrophages (TAMs) (10, 13–15). Alteration of the tumor microenvironment by the signaling pathway could either suppress or promote tumor cells. As a modulator of anti-tumor immune response, activation of cGAS-STING signaling pathway by radiation induces interferon production in colorectal cancer (16, 17). In lung cancer, STING promotes activation of lymphocytes or promotes M2 macrophage to be re-educated as M1 macrophage (18, 19). STING also enhances CD8^+^ T-cell recruitment and/or mediates APC response in glioma, head and neck carcinoma, melanoma, pancreatic cancer, and prostate cancer (4). Apart from these anti-tumor effects, STING also has a pro-tumor role. Chronic STING activation leading to chronic SASP and chronic type I IFN signaling can cause immune suppression and promote metastasis (20, 21).

Tumor cells must evade or adapt to the cGAS-STING signaling pathway to proliferate and survive (22). This facilitates STING protein as a putative target for cancer therapy. Classic cancer therapies, such as radiation and chemotherapy, increase DNA damage to promote tumor clearance through the cGAS-STING signaling pathway (23). However, persistent stimulation could lead to resistance and diminishing effect (8). STING has been considered as an ideal adjuvant for immune checkpoint inhibitors due to its ability to improve T-cell response via type I IFN (20). Recent studies have shown that activation of STING increases expression of PD-1 pathway components in murine carcinomas, and the combination of STING agonist with CTLA-4 and PD-1 antibodies has a survival advantage in mouse tumor models (24, 25). STING agonists are also being designed for tumor vaccines and chimeric antigen receptor (CAR) T-cell therapies (26). When developing STING agonists, the tumor microenvironment should be vastly considered since prolonged usage of STING agonist could potentially lead to a pro-tumor effect (27).

The association between STING and survival outcome has shown variability in previous studies. Some studies have suggested that the expression level of STING or STING-related genes is related to a favorable prognosis, while other studies indicate that STING expression in tumor or stromal cells is associated with the worst prognosis (28–33). A pan-cancer study utilizing data from The Cancer Genome Atlas (TCGA), however, has suggested that STING mRNA levels are not a prognostic factor for most tumor types (34). This may reflect the ambivalent nature of the cGAS-STING signaling pathway, where both anti-tumor and tumor-promoting mechanisms could be expected (1, 22). Nevertheless, a systematic review of immunoexpression of STING across solid tumors has not been addressed.

In this study, therefore, we conducted a meta-analysis to determine the prognostic significance of STING-positive cells in solid tumors. The aim of this study is to clarify the prognostic role of STING expression as a biomarker across multiple tumors and to verify which cell components (tumor cell or stromal cell) and survival outcome could be represented as a prognostic marker in human solid malignancies.

## 2 Materials and methods

### 2.1 Publication search strategy

Three electronic databases, PubMed, Web of Science, and Embase, were searched for relevant publications until 1 June 2023. Search terms were (“STING” or “STING1” or “Stimulator of interferon genes” or “TMEM173), (“prognosis” or “prognostic” or “outcome” or “survival”), and (“tumor” or “carcinoma” or “malignancy” or “malignant tumor”). Each source and collection period of tumor samples was carefully recorded to avoid studies with identical patient populations. Investigation was conducted by two pathologists (YK and GHK), and consensus was reached for any discrepancies for the cases. This review was reported under the Preferred Reporting Program for Systematic Reviews and Meta-Analysis (PRISMA). The protocol was registered on the International Prospective Register of Systematic Reviews (registration number: CRD42023427027).

### 2.2 Inclusion and exclusion criteria

Inclusion criteria for systematic review were as follows: (1) studies published in English; (2) original articles that report the correlation between immunohistochemical expression of STING and outcome; and (3) studies that offer hazard ratio (HR) and 95% confidence intervals (CIs) directly from the main article or supplementary material or studies that provide alternative values that could estimate HR and 95% CIs. The following studies were excluded from the systematic review: (1) studies that were from abstracts of conferences, reviews, and comments; (2) studies that were based on xenograft models or human hematopoietic or lymphoid malignancies; and (3) studies that had insufficient pathological or survival data that led to unavailability to extract HR and 95% Cis.

### 2.3 Data extraction and quality assessment

Two reviewers independently collected data from eligible articles according to the criteria. The Newcastle–Ottawa Scale (NOS) was used to evaluate the quality of each study. Studies with a score ≥6 out of 9 were defined as qualified for further analysis. The following parameters were extracted from each study: surname of first author, year of publication, primary organ, type of cancer, cell type (tumor cell or stromal cell) assessed for STING expression, cutoff method for STING-positivity, type of outcome, and number of analyzed patients. HR and 95% CIs for overall survival (OS), disease-free survival (DFS), and recurrence-free survival (RFS) were initially extracted from univariate Cox analysis when available. If HR and 95% CIs were not available, they were extracted from multivariate Cox analysis or estimated from Kaplan–Meier curves using Engauge Digitizer software (version 9.8, http://markummitchell.github.io/engauge-digitizer/) and methods provided by Tierney et al. (35).

### 2.4 Statistical analysis

The correlation between STING expression of tumor cell or stromal cell of solid tumor and prognosis of patients was measured via meta-analysis. Meta-analysis of OS and DFS/RFS was conducted with R programming (version 4.3.0) packages “meta” and “dmetar”. Cochran’s *Q* and *I* ^2^ were used to determine statistical heterogeneity. Random-effects model was used due to significant heterogeneity in most pooled analysis (*I* ^2^ > 50%). Subgroup analysis was performed to explore sources of heterogeneity. To assess publication bias, a graphical funnel plot and Egger’s test were evaluated. Sensitivity analysis was conducted to find out the effect of a single study to the pooled analysis. Statistical significance was set at *p* < 0.05.

## 3 Results

### 3.1 Literature search and study characteristics

**Figure 1:**
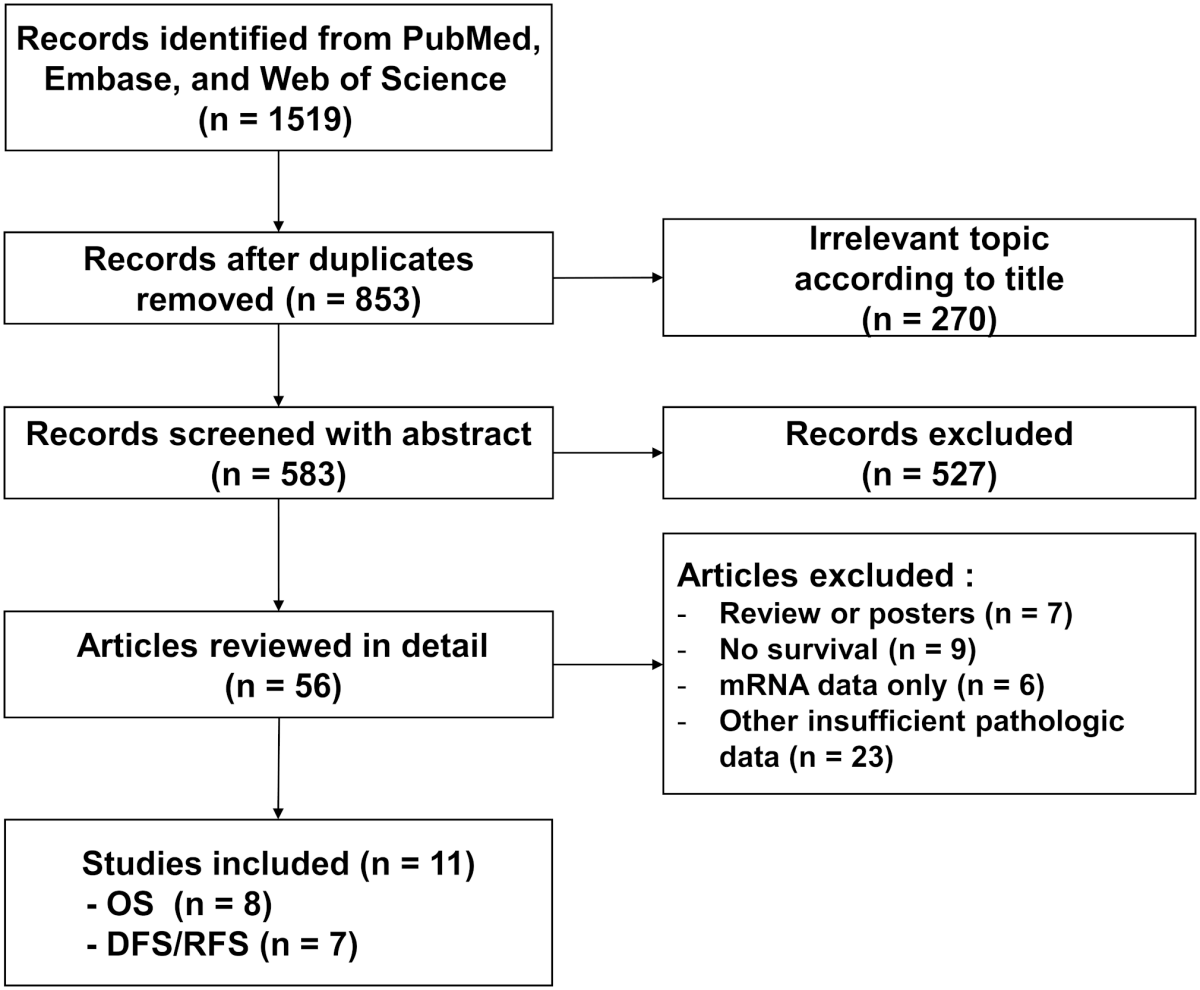
Flowchart of literature search and study selection.

A total of 853 studies were found from PubMed, Embase, and Web of Science after removing duplicate records. The detailed process of study selection is depicted in **Figure 1**. A total of 842 studies were excluded because they are not a complete article, they are irrelevant to the current subject, they lack information about STING expression and survival outcome, or they only have mRNA data about STING. In the end, 11 studies fulfilled the selection criteria and were included for the meta-analysis (30, 31, 33, 35–42). All eligible studies were retrospective and included STING expression of tumor cells (**Table 1**). Four studies also included STING expression in stromal cells (33, 39, 41, 43). Among these studies, the study by Biesaga et al. examined STING expression of all stromal cells while three other studies examined its expression for immune cells only. Two studies included two independent cohorts, although each cohort included carcinoma from identical organs (30, 37).

**Table 1:** Characteristics of studies eligible for examination of STING in tumor cells and stromal cells.

Study	Year	Availability	Sources	Survival	Total *N*	Tumor cell positivity (%)	Stromal cell positivity (%)	Organ	Subtype	Stages	Cutoff	NOS
Biesaga et al. [37]	2022	Cox	Univariate	OS/DFS	77	71.4	71.4	Head and neck	SQ	I–IV	NA	7
Chon et al. [30]	2019	Cox	Multivariate	OS/RFS	225	50.2	NA	Colorectum	AD	I–IV	Median	8
Kol et al. [31]	2021	Cox	Univariate	DFS	251/255	50.4	NA	Cervix	AD,SQ	I–IV	Median	7
Lohinai et al. [32]	2021	Cox	Univariate	OS	421	55.6	NA	Lung	AD,SQ	I–IV	H-score (>50)	7
Marletta et al. [25]	2023	Cox	Multivariate	RFS	146	36.3	NA	Kidney	RCC	I–IV	H-score (>5)	8
Parkes et al. [33]	2021	Cox	Univariate	RFS	156	50.0	50.0	Breast	IDC, ILC, mixed	Early stage	Median	7
Song et al. [34]	2017	Cox	Univariate	OS	217	35.5	NA	Stomach	Gastric cancer	I–IV	Modified H-score (>6)	7
Sun et al. [24]	2021	Cox	Univariate	OS	76/210	53.1	NA	Stomach	Gastric cancer	I–IV	Modified H-score (>6)	8
Wang et al. [27]	2017	Cox	Univariate	OS/DFS	112	50.9	50.9	Esophagus	SQ	I–IV	Median	8
Zhang et al. [35]	2021	Cox, KM	Uni-/multivariate	OS/DFS	112	42.9	67.9	Liver	HCC	NA	Median	7
Zhong et al. [36]	2018	KM	Univariate	OS	87	9.2	NA	Colorectum	AD	I–III	Any expression	8

* NOS, Newcastle–Ottawa Scale; KM, Kaplan–Meier curve; OS, overall survival; DFS, disease-free survival; RFS, recurrence-free survival; SQ, squamous cell carcinoma; AD, adenocarcinoma; RCC, renal cell carcinoma; IDC, invasive ductal carcinoma; ILC, invasive lobular carcinoma; HCC, hepatocellular carcinoma.

### 3.2 STING expression in tumor cells and survival

**Figure 2:**
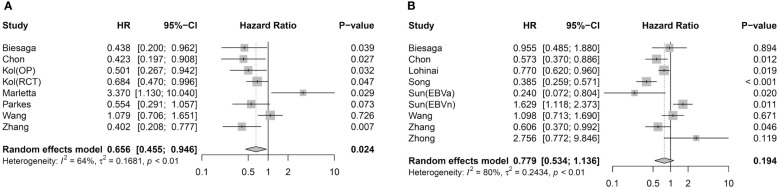
Association between STING expression in tumor cells and survival outcome. **(A)** Forest plot for DFS/RFS. **(B)** Forest plot for OS.

The meta-analysis between STING expression in tumor cells and the prognostic value of DFS/RFS involved seven articles with eight effect sizes (ESs) (**Figure 2A**). Pooled analysis demonstrated that STING-positive tumor cells are associated with better DFS/RFS in solid malignancies (HR = 0.656, 95% CI = 0.455–0.946, *p* = 0.024). Eight studies with nine ESs included the correlation between STING expression in tumor cells and OS (**Figure 2B**). Unlike DFS/RFS, OS was not significantly associated with STING expression in tumor cells (HR = 0.779, 95% CI = 0.534–1.136, *p* = 0.194).

### 3.3 STING expression in stromal cells and survival

**Figure 3:**
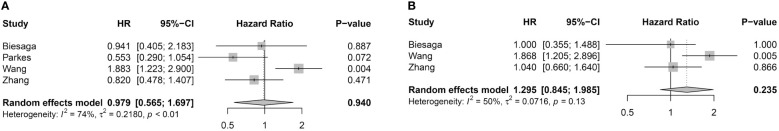
Association between STING expression in stromal cells and survival outcome. **(A)** Forest plot for DFS/RFS. **(B)** Forest plot for OS.

Four studies were available for pooled analysis of STING expression in stromal cells and DFS/RFS (**Figure 3A**). Analysis did not show significant correlation between STING-positive immune cells and DFS/RFS (HR = 0.979, 95% CI = 0.565–1.697, *p*-value = 0.940). Pooled analysis of OS also had no association with STING expression in stromal cells (HR = 1.295, 95% CI = 0.845–1.985, *p* = 0.235) (**Figure 3B**).

### 3.4 Subgroup analysis

Subgroup analysis was conducted for expression of STING in tumor cells (**Table 2** and **Supplementary Figure S1**). High STING expression in tumor cells was associated with good DFS (HR = 0.622, 95% CI = 0.428–0.903, *p* = 0.012) but not with RFS (HR = 0.868, 95% CI = 0.260–1.057, *p* = 0.818). Pooled analysis of STING-positive tumor cells from univariate Cox analysis was associated with better DFS/RFS (HR = 0.667, 95% CI = 0.482–0.923, *p* = 0.015) but those from multivariate analysis were not associated (HR = 0.783, 95% CI = 0.211–2.906, *p* = 0.714). Inversely, STING-positive tumor cells were associated with better OS for multivariate studies (HR = 0.587, 95% CI = 0.424–0.814, *p* = 0.001), but not univariate studies (HR = 0.853, 95% CI = 0.517–1.408, *p* = 0.535). STING-positive tumor cells of the gastrointestinal system was associated with improved DFS/RFS (HR = 0.566, 95% CI = 0.330–0.971, *p* = 0.039), but not with OS (HR = 0.576, 95% CI = 0.185–1.796, *p* = 0.342). Correlation between OS and STING expression of tumor cells was not significant despite considering the difference in cutoff method (HR = 0.730, 95% CI = 0.481–1.109, *p* = 0.140 and HR = 0.642, 95% CI = 0.297–1.388, *p* = 0.260 for median and H-score, respectively).

**Table 2:** Subgroup analysis with STING expression in tumor cells.

Type of survival	Subgroup	No. of studies	Hazard ratio	Lower limit of 95% CI	Upper limit of 95% CI	*p*-value
RFS/DFS	DFS	5	0.622	0.428	0.903	0.012*
RFS/DFS	RFS	3	0.868	0.260	1.057	0.818
RFS/DFS	Univariate	5	0.667	0.482	0.923	0.015*
RFS/DFS	Multivariate	3	0.783	0.211	2.906	RFS/DFS
RFS/DFS	GI system	4	0.566	0.330	0.971	0.039*
OS	Univariate	7	0.853	0.517	1.408	0.535
OS	Multivariate	2	0.587	0.424	0.814	0.001*
OS	GI system	7	0.576	0.185	1.796	0.342
OS	Median	3	0.730	0.481	1.109	0.140
OS	(Modified) H-score	4	0.642	0.297	1.388	0.260

*p < 0.05.

### 3.5 Evaluation of publication bias and sensitivity analysis

**Figure 4:**
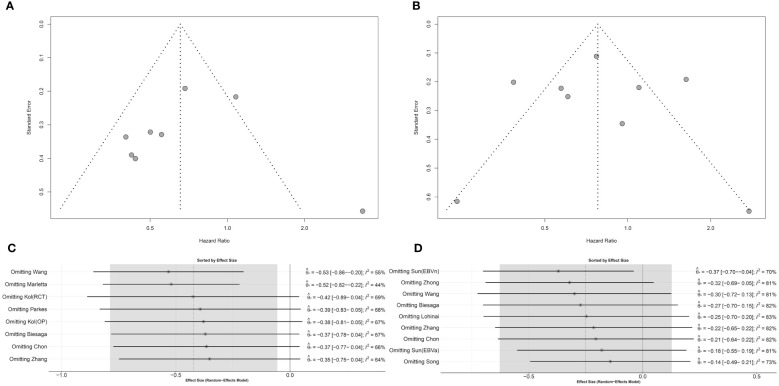
Publication bias and sensitivity analysis for STING expression in tumor cells. **(A)** Funnel plot for DFS/RFS. **(B)** Funnel plot for OS. **(C)** Sensitivity analysis for DFS/RFS. **(D)** Sensitivity analysis for OS.

Publication bias in the eligible studies was evaluated by a graphical funnel plot and Egger’s test. Studies with DFS/RFS and tumor cells showed potential asymmetry in the funnel plot, but according to Egger’s test, a significant publication bias was not suspected (*p* = 0.828) (**Figure 4A**). Studies with OS and tumor cells showed symmetric distribution (*p* = 0.999 in Egger’s test) (**Figure 4B**). Omitting individual ES in DFS/RFS and OS has demonstrated that robustness of the pooled analysis could be limited (**Figures 4C, D**). Identical analyses were shown for the correlation between STING expression in stromal cells and DFS/RFS or OS (**Supplementary Figure S2**).

## 4 Discussion

The cGAS-STING signaling pathway, triggered by sensoring dsDNA, activates kinases IKK and TBK1, leading to a cascade that transcripts pro-inflammatory cytokines including type I IFN, which recruits cytotoxic T cells and NK cells for tumor cell clearance (44). Consequently, expression of STING has been suggested as a therapeutic target as well as a prognostic biomarker in solid tumors. However, the evaluation of STING immunoexpression in various cell types, such as tumor cells, T cells, and macrophages, has shown varying degrees of association with survival outcome. This study, for the first time, explores the correlation between STING-positive tumor cells or stromal cells and prognosis in solid tumors through meta-analysis.

In a pooled analysis, STING expression of tumor cells was associated with improved DFS/RFS. Subgroup analysis revealed that excluding studies from multivariate analysis did not impact the overall association between STING expression and DFS/RFS. A subgroup analysis of the gastrointestinal system maintained the correlation between STING expression in tumor cells and DFS/RFS. However, a subgroup analysis with a smaller number of studies (equal to or fewer than three studies) was determined insignificant with DFS/RFS. In the pooled analysis for stromal STING, the correlation with survival was not significant. However, it should be emphasized that only a small number of studies were obtained compared with that of tumoral STING (four and three for DFS/RFS and OS, respectively).

STING expression in tumor cells was largely unassociated with OS in overall pooled analysis and in subgroup analysis. These results align with previous studies analyzing TCGA data, in which mRNA expression of STING was mostly unrelated to OS regardless of carcinoma type (34). Intriguingly, high STING mRNA expression was associated with worse OS in renal cell carcinomas, and a similar trend has been observed for immunohistochemical STING expression in renal cell carcinoma (31). These similarities suggest that STING mRNA in renal carcinoma strongly recapitulates STING expression in tumor cells, rather than that in stromal cells despite estimated tumor purity of renal cell carcinomas in TCGA only being intermediate (45).

Currently, there is no clear explanation for the discrepancies shown between DFS/RFS and OS in tumor cells. STING activity has been identified as a source of suppressor of spontaneous outbreak from disseminated cancer cells in lung adenocarcinoma (46). Expression of STING enhances tumor cell clearance by T cells and NK cells, thereby inhibiting metastasis and tumor relapse. Considering that a relapse of carcinoma would directly affect DFS and RFS but not OS per se, it could be hypothesized that the mechanism of immune recognition and evasion associated with STING during metastasis contributes to the difference in results observed between the survival outcomes.

STING is a pivotal regulator of cancer immunity and initiates innate immune response (47). As an upstream signaling molecule, various methods for the evaluation of active STING could be considered. In this study, immunohistochemical expression of STING was considered as a putative marker for STING activation. A more direct method would be examining phosphorylated STING, which consequently activates TBK1 and recruits IRF3, but to the best of our knowledge, a study that includes immunohistochemical staining of phospho-STING in tumors has yet been published (48). Another way to evaluate STING activation would include assessment of downstream effector molecules. STING-related gene signatures were associated with prognosis in breast, prostate, and colorectal cancers (28, 29, 32). Therefore, developing alternative protein markers alongside STING could provide a more comprehensive understanding of STING activation and its functional implications even in cases when upstream signaling pathway is compromised by genetic alterations.

There are several limitations in this study. The primary tumor sites analyzed for DFS/RFS and OS are not identical and, therefore, may not have an identical impact compared with when all organs are matched. Subgroup analysis for gastrointestinal systems was identical to the overall pooled analysis for both survival outcomes. Nevertheless, STING expression in tumor cells could have an anti-tumor and a pro-tumor effect according to specific tumor context, and therefore, trying to analyze STING expression of all solid malignancy in a uniform manner might undermine the distinct role of the cGAS-STING signaling pathway for each tumor. Another limitation involves the sensitivity analysis revealing limitations in robustness. However, there was no significant publication bias despite the relatively small number of studies available for each pooled analysis of tumor cells. Lastly, our analysis of STING was restricted to cytoplasmic and membranous expression, with only one study describing subcellular location other than cytoplasm and membrane as a region associated with prognosis (39).

In conclusion, this meta-analysis demonstrated that STING expression in tumor cells is associated with improved DFS/RFS but not OS, while in small available studies, STING expression in stromal cells has no association with survival outcome when evaluating malignancies from various organs. Further studies should investigate whether the prognostic value of STING expression in tumor cells can serve as a viable target for predicting therapeutic response and guiding personalized treatment strategies in solid tumors.

## Data availability statement

The original contributions presented in the study are included in the article. Further inquiries can be directed to the corresponding author.

## Author contributions

YK and GHK: protocol, data collection, and funding acquisition; YK: conceptualization and writing the first manuscript; GHK: reviewing the manuscript and supervision; HJ: quality assessment; N-YC and LJ: figure legends. All authors contributed to the article and approved the submitted version.
