# Supplementary figures and images for: Prognostic significance of STING expression in solid tumor: a systematic review and meta-analysis
Source: Front Oncol. 2023 Aug 29;13:1244962. doi: 10.3389/fonc.2023.1244962 (PMC10497868; doi:10.3389/fonc.2023.1244962)

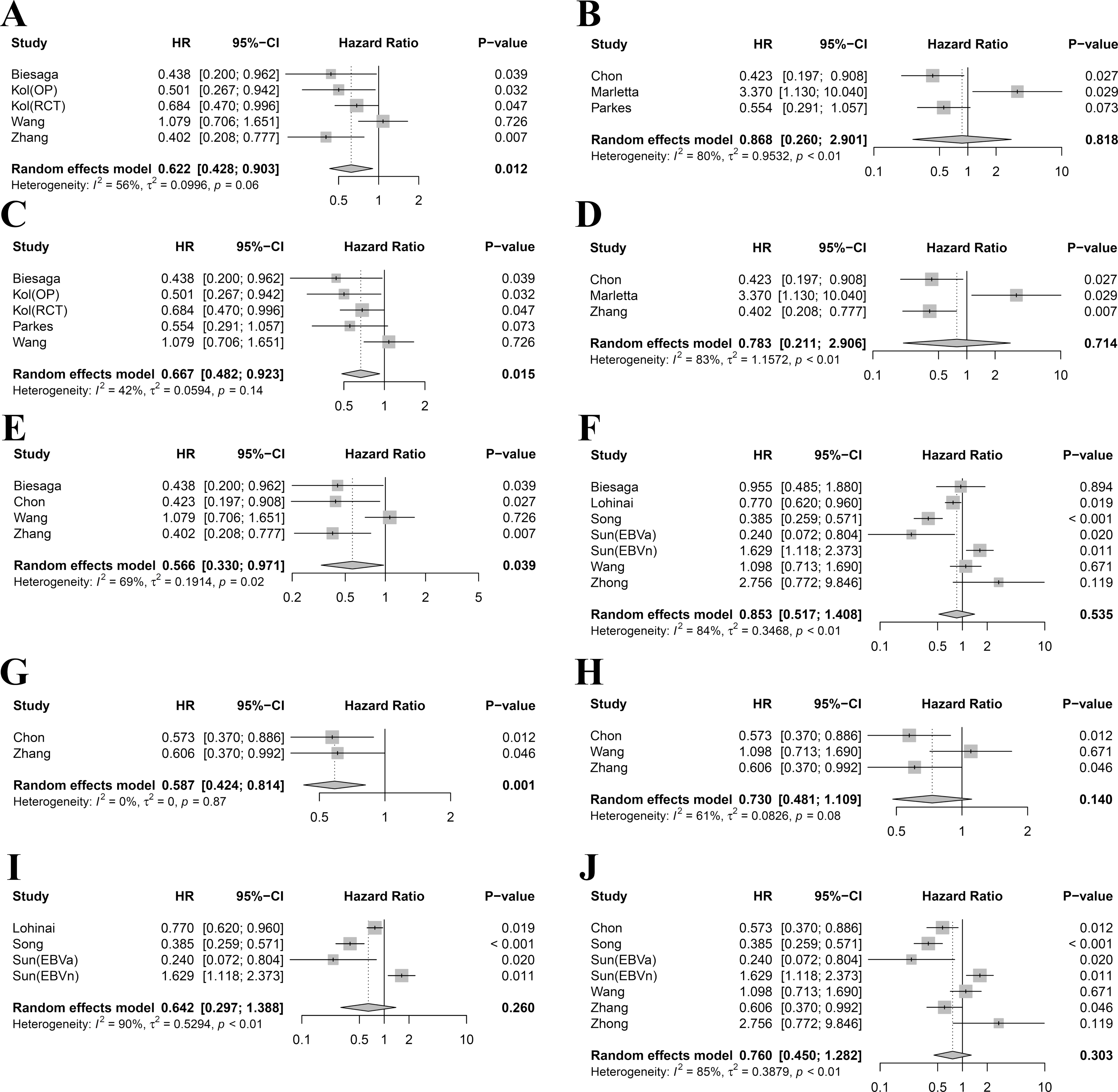

Supplement: Supplementary file 1 [file Image_1.tif]

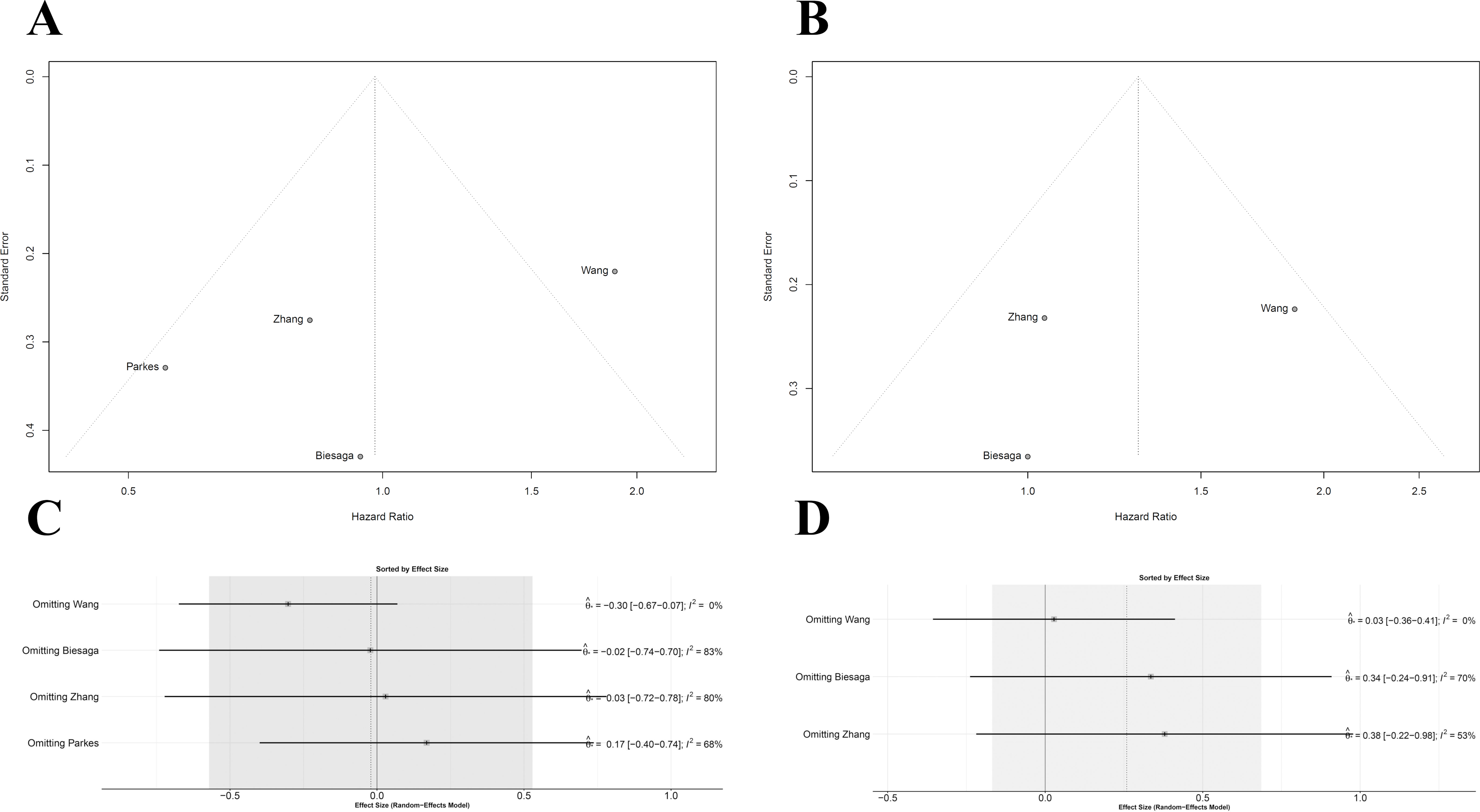

Supplement: Supplementary file 2 [file Image_2.tif]
